# Culturable bacteria from an Alpine coniferous forest site: biodegradation potential of organic polymers and pollutants

**DOI:** 10.1007/s12223-020-00825-1

**Published:** 2020-09-25

**Authors:** Tanja Berger, Caroline Poyntner, Rosa Margesin

**Affiliations:** Institute of Microbiology, University of Innsbruck, Technikerstrasse 25, 6020 Innsbruck, Austria

## Abstract

The potential of the culturable bacterial community from an Alpine coniferous forest site for the degradation of organic polymers and pollutants at low (5 °C) and moderate (20 °C) temperatures was evaluated. The majority of the 68 strains belonged to the phylum Proteobacteria (77%). Other strains were related to Bacteroidetes (12%), Alphaproteobacteria (4%), Actinobacteria (3%), and Firmicutes (3%). The strains were grouped into 42 different OTUs. The highest bacterial diversity was found within the phylum Bacteroidetes. All strains, except one, could grow at temperatures from 5 to 25 °C. The production of enzyme activities involved in the degradation of organic polymers present in plant litter (carboxymethyl cellulose, microgranular cellulose, xylan, polygalacturonic acid) was almost comparable at 5 °C (68%) and 20 °C (63%). Utilizers of lignin compounds (lignosulfonic acid, lignin alkali) as sole carbon source were found to a higher extent at 20 °C (57%) than at 5 °C (24%), but the relative fractions among positively tested strains utilizing these compounds were almost identical at the two temperatures. Similar results were noted for utilizers of organic pollutants (n-hexadecane, diesel oil, phenol, glyphosate) as sole carbon source. More than two-thirds showed constitutively expressed catechol-1,2-dioxygenase activity both at 5 °C (74%) and 20 °C (66%). Complete phenol (2.5 mmol/L) degradation by strain *Paraburkholderia aromaticivorans* AR20-38 was demonstrated at 0–30 °C, amounts up to 7.5 mmol/L phenol were fully degraded at 10–30 °C. These results are useful to better understand the effect of changing temperatures on microorganisms involved in litter degradation and nutrient turnover in Alpine forest soils.

**Electronic supplementary material:**

The online version of this article (10.1007/s12223-020-00825-1) contains supplementary material, which is available to authorized users.

## Introduction

Climate change is increasing surface temperatures worldwide and is one of the largest challenges facing our planet. Besides a number of physical and chemical alterations, such as increasing soil temperatures, carbon dioxide levels, or nitrogen deposition, climate change will affect the structure, diversity, and abundance of soil microbial communities. Knowledge on the response of soil microorganisms to environmental changes is essential to understand and predict ecosystem processes (Li et al. 2014). Alpine mountain systems are especially vulnerable. The European Alps already experienced an increase in the annual minimum temperature of ca. 2 °C during the twentieth century (Gobiet et al. 2014).

The largest pool of carbon on Earth is stored in forest soils. Since forests are known to act as carbon sinks (Baldrian 2017), they play an important role in global carbon cycling and consequently affect climate. The main carbon reservoir in forests is soil organic matter (SOM). Soil microorganisms play a crucial role in the transformation and decomposition of SOM (Žifčáková et al. 2017). In earlier studies, we examined the effects of altitude and season on the microbial diversity and activity along an Alpine elevation gradient (França et al. 2016; Siles et al. 2016). The functional characterization of the soil microbial communities demonstrated that soil microorganisms at the coniferous forest site (1724–1737 m above sea level) have a higher resistance to environmental changes (contents of SOM and nutrients, soil temperature, soil pH) than those from a deciduous forest site at lower altitude (Siles and Margesin 2017). At this site, genes related to cellulose (mainly genes encoding cellobiase and exoglucanase) and lignin (phenol oxidase) degradation and genes involved in bioremediation, such as for the degradation of aromatic compounds, herbicides, and pesticides, were found. The coniferous forest site was also characterized by significantly increased levels of genes involved in the degradation of both labile (starch) and recalcitrant (cellulose, pectin, and lignin) carbon compounds in autumn compared with spring (Siles and Margesin 2017).

It is recognized that a combination of culture-dependent and culture-independent methods is successful in exploring the abundance and diversity of microorganisms in environmental samples. However, culture-dependent methods are still required to understand microbial functions, physiology, adaptation mechanisms, and community dynamics in their environment and to be able to detect and use microorganisms and their associated bioproducts for biotechnological applications (Gebbie et al. 2020). Low-temperature environments present a large resource for the discovery of useful microorganisms with a wide range of potential biotechnological applications (Collins and Margesin 2019).

To gain further insights into the bacterial activity at the above-mentioned and well-studied coniferous forest site R, we evaluated the biodegradation potential of the culturable bacterial community isolated earlier in autumn (França et al. 2016). We characterized 68 strains with regard to the production of enzymes involved in the degradation of organic compounds and with regard to the utilization of lignins and organic pollutants as sole carbon source. To reveal the phenotypes adapted to the site-specific conditions, bacterial activities were evaluated at low (5 °C) and moderate (20 °C) temperatures.

## Materials and methods

### Sampling and isolation of bacteria

The sampling site (coniferous Alpine forest) has been described in detail by França et al. (2016). Briefly, the site R (N 46° 35′ 16.2″, E 11° 26′ 4.9″) is located 7 km north of Bozen/Bolzano below the Rittner Horn at an altitude of 1724–1737 m above sea level. The pedogenic substratum consists of rhyolite (quartz-porphyry) and the soil was classified as haplic podzol (FAO). The site consists of coniferous forest close to the timber line, dominated by *Picea abies*, *P. cembra*, *Larix decidua*, and *Vaccinium myrtillus*. The climate is subalpine-continental with an annual precipitation of 1000 mm, a mean annual air temperature of 4.0 °C, a mean annual soil temperature of 4.3 °C, a minimum annual soil temperature of 1.9 °C, and a maximum annual soil temperature of 6.1 °C (França et al. 2016).

Soil samples were collected from this site in late autumn (15 October 2014). Immediately after sampling, soil samples were transported in cooled boxes to the laboratory, sieved (< 2 mm), and immediately analyzed for culturable microorganisms. The determination of soil characteristics demonstrated an acidic soil pH (3.4). Contents of humus, TOC (total organic carbon), and total N were 53%, 40%, and 1.4%, respectively. The C/N ratio was 23 (França et al. 2016). The 68 bacterial strains described in this study were isolated on R2A agar supplemented with cycloheximide (400 μg/mL) at 20 °C (França et al. 2016) and stored at − 80 °C in R2A broth supplemented with 15% (w/v) glycerol.

### Identification and phylogenetic analysis of culturable bacteria

Bacterial genomic DNAs were extracted by microwave lysis (Sánchez-Hidalgo et al. 2012). The primers 27F (5′-AGAGTTTGATCCTGGCTC-3′) and 1541R (5′-AAGGAGGTGATCCAGCCGCA-3′) (Lane 1991) were used for partial 16S rRNA gene amplification. Each 50 μL PCR reaction contained 5 μL PCR buffer (10x) (BIORON GmbH, Germany), 1 μL MgCl_2_ (100 mmol/L) (BIORON GmbH), 1 μL dNTPs (10 mmol/L each) (Sigma, USA), 2 μL each forward and reverse primers (10 μmol/L) (Eurofins Genomics, Germany), 0.5 μL Taq polymerase (5 U/μL) (BIORON GmbH), 4 μL DNA solution, and 34.5 μL H_2_O. The thermal cycling program was as follows: (i) 5 min at 95 °C; (ii) 35 cycles of 30 s at 95 °C, 30 s at 52 °C, and 1 min at 72 °C; and (iii) a final elongation step of 10 min at 72 °C. 16S rRNA PCR gene products were visualized on an ethidium bromide–stained agarose gel, purified using GENEJET PCR purification kit (Thermo Fisher Scientific Inc., USA) according to the manufacturer’s instructions and Sanger sequenced (Microsynth AG, Switzerland).

**Fig. 1:**
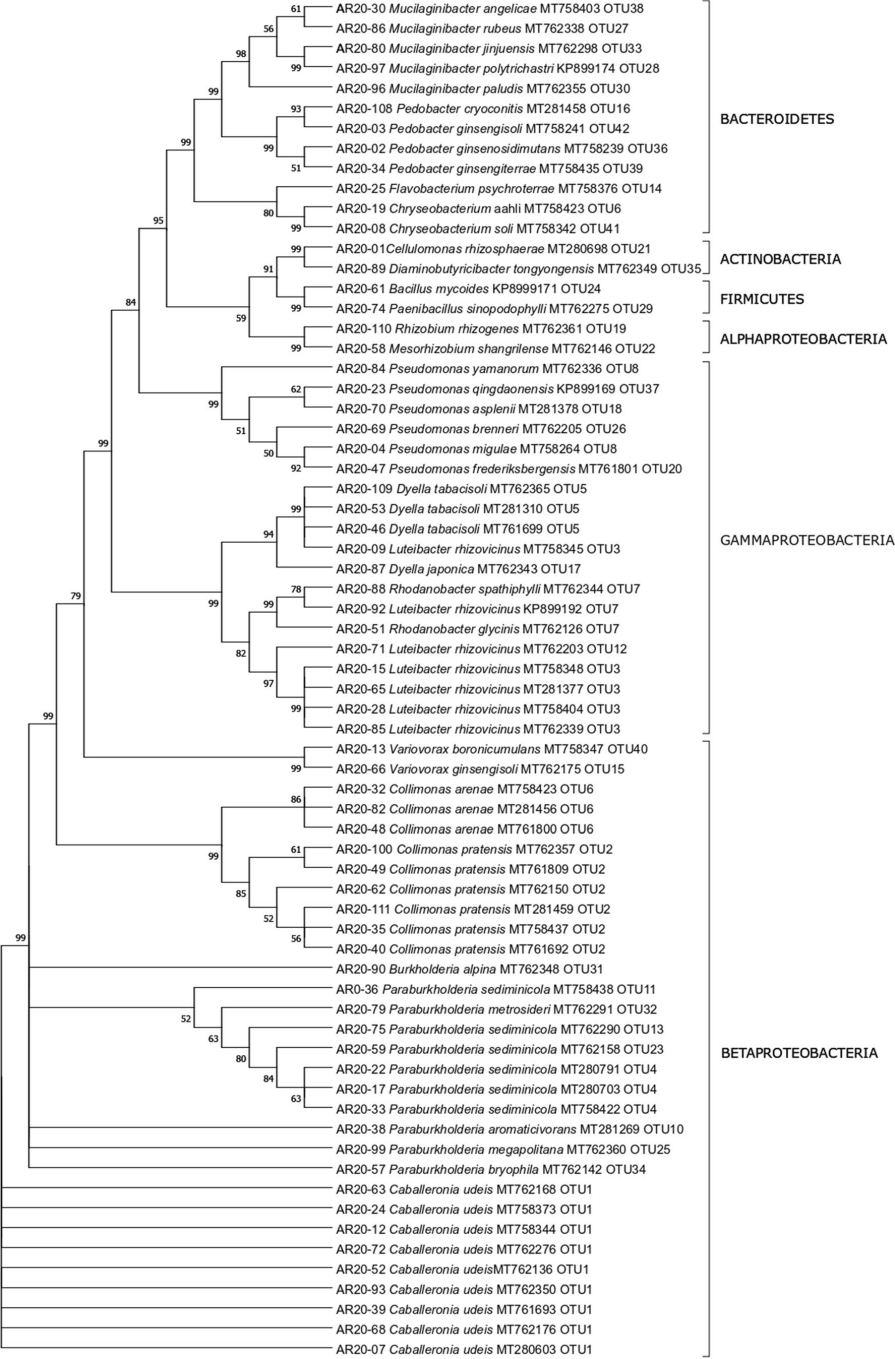
Bootstrap consensus tree with the highest log likelihood (− 7828.05) using maximum likelihood method and the general time reversible model is shown. Numbers next to the branches show percentage of trees in which the associated taxa clustered and different classes and phyla are marked on the right. The species resulting from EzTazon-e Database, OTUs, and Genbank Accession numbers are given next to the species number. The tree is drawn to scale, with branch lengths measured in the number of substitutions per site

The sequences were manually edited using the software MEGA ver. X and the nearest phylogenetic neighbors were determined for each strain using the EzTazon-e Database (Kim et al. 2012). This information was used to describe the taxonomic bacterial diversity. The sequences were then clustered into operational taxonomic units (OTUs) at 99% identity using average neighbor algorithm with the software Mothur v.1.39.3 (Schloss et al. 2009). All 16S rRNA gene sequences were deposited in GenBank NCBI under the accession numbers shown in Fig. 1 and Table S1.

Phylogenetic trees of the 68 16S sequences (Fig. 1) and 42 reference strains resulting from the OTU clustering (Fig. S1) were constructed to visualize their relationships using Mega X (Kumar et al. 2018). Maximum composite likelihood approach was used and after testing for the best model, the general time reversible model (Nei and Kumar 2000) was chosen for Fig. 1 and the Kimura 2-parameter model (Kimura 1980) was chosen for Fig. S1. A discrete gamma distribution (5 categories (+G, parameter = 0.4770 Fig. 1/0.5792 Fig. S1)) was used and some sites were evolutionary invariable ([+I], 26.40% sites Fig. 1/26.94% sites Fig. S1). The partial deletion option was chosen, and bootstrapping values were calculated using the neighbor joining method (resampling size *n* = 2000).

### Growth temperature range of bacterial strains

Suspensions of bacterial cells (pre-grown on R2A) in 0.9% NaCl were used to inoculate R2A agar plates that were incubated at 0, 5, 10, 15, 20, 25, 30, and 35 °C, using two replicates per strain and temperatures. Growth was monitored over an incubation time of 21 days.

### Screening for enzyme activities

Activities of cellulases, xylanase, and pectate lyase of the 68 strains were evaluated on R2A agar plates supplemented with carboxymethyl cellulose (CMC, Sigma C-5678), microgranular cellulose (micCell, Sigma C-6413), xylan (from larchwood, Sigma X-3875), and polygalacturonic acid (PGA, from orange, Sigma P-7276), using two replicates per strain and compound. For each compound, a concentration of 5 g/L (0.5% w/v) was used. Inoculated plates without these compounds were used as negative controls. After incubation at 5 °C (14 days) and 20 °C (7 days), plates containing celluloses and xylan were flooded with Congo red solution (1 mg/mL) for 15 min followed by flooding with 1 mol/L NaCl for 15 min and rinsing with 1 mol/L NaCl (Li et al. 2016). A positive reaction was noted when transparent zones around the colonies were detected. PGA-containing plates were flooded with 1 mol/L calcium chloride for 20 min; a positive reaction was noted when distinct cloudy halos appeared around enzyme-producing colonies (Margesin et al. 2005).

For the evaluation of constitutively expressed catechol dioxygenase activities, the 68 bacterial strains were grown at 5 °C and at 20 °C on R2A agar plates. Cells were collected from the agar plate and suspended in 0.9% NaCl; the A_600_ value of all cell suspensions was adjusted to 1.5 with 0.9% NaCl (= standardized cell suspensions). By using these standardized cell suspensions, it was possible to compare the activities of the strains. Catechol-1,2-dioxygenase (C1,2D) and catechol-2,3-dioxygenase (C2,3D) were quantified spectrophotometrically as described (Nakazawa and Nakazawa 1970; Nozaki 1970; Margesin et al. 2013). The product formation of cis,cis-muconic acid from catechol (260 nm) indicated C1,2D, while the formation of 2-hydroxymuconic semialdehyde (375 nm) was indicative of C2,3D. Three replicates were used per strain and enzyme.

### Screening for utilization of lignin as sole carbon source

Suspensions of bacterial cells in 0.9% NaCl were used to inoculate plates composed of mineral medium (Margesin and Schinner 1997) with purified agar, without yeast extract. The medium contained either lignin sulfonic acid (LSS) (Roth 8999, water soluble, 2 g/L) or lignin alkali (Aldrich 370959, insoluble in water, 5 g/L) the sole carbon source. Inoculated plates without these compounds were used as negative controls. Plates were incubated up to 21 days at 5 °C and at 20 °C, using two replicates per strain and compound, and growth was monitored regularly.

The LSS-utilizing strains AR20-38 and AR20-07 were further tested in liquid culture in 100-mL Erlenmeyer flasks containing 20 mL of mineral medium to determine the effect of temperature (0–30 °C) and LSS concentration (2, 4, 6, 8, 10, 15 g/L) on growth. Growth (A_600_) was monitored regularly.

### Screening for utilization of organic pollutants as sole carbon source

Suspensions of bacterial cells in 0.9% NaCl were used to inoculate mineral medium (see above) agar plates containing purified agar. The plates were amended with one of the following compounds as the sole carbon source: n-hexadecane (50 μL per plate, supplemented via the vapor phase), diesel oil (40 μL per plate, supplemented via the vapor phase), phenol (2.5 mmol/L = 235 mg/L), and glyphosate (Roundup© 1 g/L). Inoculated plates without these compounds were used as negative controls. Plates were incubated up to 14–21 days at 5 °C and at 20 °C, using two replicates per strain and compound, and growth was monitored regularly.

The phenol-utilizing strain AR20-38 was further tested in liquid culture in 100-mL Erlenmeyer flasks containing 20 mL of mineral medium to determine the effect of temperature (0–35 °C) and phenol concentration (2.5, 5, 7.5, 10 mmol/L) on phenol degradation. Growth (A_600_) and the residual phenol content (Allsop et al. 1993; Margesin et al. 2013) were monitored regularly. Phenol concentration was determined in culture supernatants that were filtered (0.2 μm, Minsart RC4 17821) after centrifugation. High-performance liquid chromatography (HPLC) was carried out by using a RP-18 column (5 μm × 100 mm, Lichrospher, Merck), detection at 220 nm (Shimadzu SPD-20A), and an eluent flow of 0.5 mL/min. The elution time for phenol was approx. 7.5 min. The phenol calibration curve was prepared in mineral medium.

## Results

### Culturable bacterial diversity

The phylogenetic relationship between the studied strains is shown in Fig. 1. The majority of the studied 68 strains (52 strains, 77%) belonged to the phylum Proteobacteria, with a predominance of the two classes Betaproteobacteria (60% of Proteobacteria) and Gammaproteobacteria (37% of Proteobacteria), while Alphaproteobacteria were present in a very low amount (4%) (Fig. 1, Table 1). All representatives of Betaproteobacteria belonged to the order Burkholderiales, with a predominance of the genera *Paraburkholderia* and *Caballeronia*; Xanthomonadales dominated among Gammaproteobacteria. Only 18% (12 strains) were represented by Bacteroidetes. The remaining 4 strains were related to the phyla Actinobacteria and Firmicutes (2 strains each) (Fig. 1, Table 1). Thus, the fraction of Gram-positive bacteria (6%) was very low compared with the fraction of Gram-negative bacteria (94%).

**Table 1:** Identification of the studied 68 culturable bacterial strains (*n* number of representatives)

Phylum (*n*)	Class (*n*)	OTUs (*n*)	Genera (*n*)
Actinobacteria (2)		2	*Cellulomonas* (1)
			*Diaminobutyricibacter* (1)
Firmicutes (2)		2	*Bacillus* (1)
			*Paenibacillus* (1)
Bacteroidetes (12)		12	*Mucilaginibacter* (5)
			*Pedobacter* (4)
			*Chryseobacterium* (2)
			*Flavobacterium* (1)
Proteobacteria (52)	Alphaproteobacteria (2)	2	*Rhizobium* (1)
			*Mesorhizobium* (1)
	Betaproteobacteria (31)	14	*Paraburkholderia* (10)
			*Caballeronia* (9)
			*Collimonas* (9)
			*Variovorax* (2)
			*Burkholderia* (1)
	Gammaproteobacteria (19)	10	*Luteibacter* (7)
			*Pseudomonas* (6)
			*Dyella* (4)
			*Rhodanobacter* (2)

The 68 strains were grouped into 42 different OTUs at 99% identity. The OTUs 1 and 2, classified at order level as Burkholderiales (Betaproteobacteria) and clustering 9 and 6 strains, respectively, were the most abundant ones. Twenty-six OTUs were classified as Proteobacteria, 12 as Bacteroidetes, 2 as Firmicutes, and the remaining 2 as Actinobacteria. The phylogenetic tree (Fig. S1) of the 42 reference strains obtained from the OTU analysis shows that strains from the same phylum are clustering together. The highest bacterial diversity was found within the phylum Bacteroidetes since the 12 isolates belonging to this group were grouped into 12 different OTUs (Fig. 1).

### Growth temperature range

All 68 tested strains could grow at temperatures ranging from 10 to 25 °C; only one strain was unable to grow at 5 °C (Table S1). About two-thirds (44 strains, 65%) showed growth at 0 °C. Neither all four representatives of Gram-positive bacteria nor all three representatives of OTU 4 (Betaproteobacteria, *Paraburkholderia* sp.) could grow at 0 °C. In contrast, all 9 representatives of OTU 1 (Betaproteobacteria, *Caballeronia* sp.) and OTU 2 (Betaproteobacteria, *Collimonas* ssp.) showed generally good growth at temperatures below 20 °C. Fifty-seven strains (84%) were able to grow at 30 °C; the 3 representatives of OTU 7 (Gammaproteobacteria) could not grow at this temperature. Only 2 strains showed growth at 35 °C, however, not at 40 °C. One of these two strains (*Pseudomonas* sp. AR20-23) showed growth over the whole temperature range of 0–35 °C, while the other strain (*Bacillus* sp. AR20-61) was the only one among the 68 strains unable to grow at 5 °C.

### Enzyme activities

#### Enzyme activities for the utilization of organic polymers

A total of 68 strains were tested for enzyme activities required for the utilization of organic polymers using the plate assay method. All tested 68 strains could grow (which does not mean activity) on agar plates supplemented with CMC at 5 °C and 20 °C. The supplementation of the medium with xylan, PGA, or micCell, however, resulted partly in growth inhibition, being higher at the lower incubation temperature. This was especially noted for xylan (growth inhibition of 44% and 16% of the strains at 5 °C and 20 °C, respectively) and PGA (inhibition of 26% and 6% at 5 °C and 20 °C, respectively).

However, a considerable number of strains produced the enzymes required for substrate degradation (Table S1). A slightly higher amount of strains tested positive for enzyme activities at 5 °C (68%, 46 strains) than at 20 °C (63%, 43 strains), which demonstrated the adaptation of the strains to low temperature conditions (Table 2). Among the positively tested strains (Table 3), more than one-third showed CMCase activity (35% at 5 °C, 40% at 20 °C). Avicelase activity (cellulose-1,4-β-cellobiosidase; micCell hydrolysis) was detected to a significantly higher extent at 5 °C (39% of the positive strains) than at 20 °C (19%). Generally, strains tended to utilize either CMC or micCell, only few strains were able to degrade both cellulose compounds. CMCase activity was found in all Gram-positive representatives and frequently present in members of Bacteroidetes, and Beta- and Gammaproteobacteria. About one-third (6 strains) of the 19 Gammaproteobacteria members displayed CMCase activity only at 5 °C but not at 20 °C. In contrast, micCell degradation restricted to 5 °C was detected at a frequency of ca. 20% in Beta- and Gammaproteobacteria members. The ability to degrade PGA was restricted to representatives of Gammaproteobacteria (genera *Pseudomonas* and *Luteibacter*) and was more often detected at 20 °C (19% of the positive strains) than at 5 °C (11%) (Table S1).

**Table 2:** Effect of temperature on the bacterial production of enzymes for the degradation of organic polymers and the utilization of lignins and organic pollutants as sole carbon source (100% = 68)

Activity	5 °C		20 °C	
	*n*	%	*n*	%
Enzyme activities				
CMCase	16	23.5	17	25.0
Avicelase	18	26.5	8	11.8
Xylanase	7	10.3	10	14.7
Pectate lyase	5	7.4	8	11.8
Total positive	46	67.6	43	63.2
C1,2D only	50	73.5	45	66.2
C2,3D only	0	0.0	0	0.0
C1,2D + C2,3D	12	17.6	19	27.9
Lignins as sole C source				
LSS	14	20.6	34	50.0
Lignin alkali	2	2.9	5	7.4
Total positive	16	23.5	39	57.4
Organic pollutants as sole C source				
n-Hexadecane	5	7.4	7	10.3
Diesel oil	3	4.4	17	25.0
Phenol	1	1.5	1	1.5
Glyphosate	1	1.5	14	20.6
Total positive	10	14.7	39	57.4

**Table 3:** Effect of temperature on positively tested bacteria strains able to produce enzymes for the degradation of organic polymers and to utilize lignins and organic pollutants as sole carbon source (100% = total positive)

Activity	5 °C		20 °C	
	*n*	%	*n*	%
Enzyme activities				
CMCase	16	34.8	17	39.5
Avicelase	18	39.1	8	18.6
Xylanase	7	15.2	10	23.3
Pectate lyase	5	10.9	8	18.6
Total positive	46	100.0	43	100.0
Lignins as sole C source				
LSS	14	87.5	34	87.2
Lignin alkali	2	12.5	5	12.8
Total positive	16	100	39	100
Organic pollutants as sole C source				
n-Hexadecane	5	50.0	7	17.9
Diesel oil	3	30.0	17	43.6
Phenol	1	10.0	1	2.6
Glyphosate	1	10.0	14	35.9
Total positive	10	100.0	39	100.0

Several strains produced more than one of the tested enzyme activities. One strain (*Luteibacter rhizovicinus* AR20-65, Gammaproteobacteria) displayed at 5 °C all four enzyme activities tested, while none of the strains showed this ability at 20 °C. Three strains (all of them members of Bacteroidetes: genera *Chryseobacterium*, *Mucilaginibacter*, *Pedobacter*) utilized all enzyme substrates except PGA at 20 °C. Two strains (*Pseudomonas*, *Pedobacter*) degraded three enzyme substrates at 5 °C. A higher amount of strains produced two enzyme activities (10 and 8 strains at 5 °C and 20 °C, respectively). The majority, however, expressed one enzyme activity (16 and 18 strains at 5 °C and 20 °C, respectively).

#### Catechol dioxygenases

Catechol dioxygenase activities are involved in the second step of phenol degradation and catalyze the ring cleavage of catechol. Constitutively expressed catechol dioxygenase production was evaluated by using standardized cell suspensions, which were prepared by adjusting the A_600_ values of all cell suspensions to 1.5; this way, it was possible to compare the activities of the tested strains. None of the 68 strains produced only C2,3D, while more than two-thirds showed only C1,2D both at 5 °C (74%) and 20 °C (66%) (Table 2). Among the C1,2D-positive strains, a considerable part was able to produce additionally C2,3D at 5 °C (24%) and 20 °C (42%, among them 5 of the 6 representatives of OTU2, belonging to *Collimonas pratensis*, Betaproteobacteria). All strains—except one—that produced both C1,2D and C2,3D at 5 °C were able to produce both enzymes also at 20 °C, while the reverse was not noted (i.e., production at 20 °C was not combined with production at 5 °C). C1,2D was present in almost all phylogenetic representatives; the highest frequency of C2,3D was found in Bacteroidetes representatives (Table S1). Generally, C1,2D was expressed to a higher extent than C2,3D. The majority of the strains (78%) showed activities of C1,2D in the range of 1–10 U/A_600_ at 5 °C and at 20 °C, while C2,3D activity was generally 10-fold lower.

### Utilization of lignin as sole carbon source

The 68 strains were tested for their ability to utilize LSS or lignin alkali as sole carbon source on agar plates. A significantly higher fraction was able to utilize these lignin compounds at 20 °C (39 strains, 57%) than at 5 °C (16 strains, 24%) (Table 2, Table S1). However, among these positive strains (Table 3), the relative fractions utilizing the two lignin compounds (87% utilized LSS, 13% lignin alkali) were almost identical at 5 °C and 20 °C. Independent of the cultivation temperature, LSS was preferred by a significantly higher amount of strains compared with lignin alkali. Especially representatives of the order Burkholderiales (Betaproteobacteria) were found among LSS utilizers. In contrast, most of lignin alkali utilizers belonged to the genus *Pseudomonas* (Gammaproteobacteria). Only 1 strain (*Pseudomonas* AR20-70) was able to grow with both compounds as sole carbon source at both temperatures (Table S1).

**Fig. 2:**
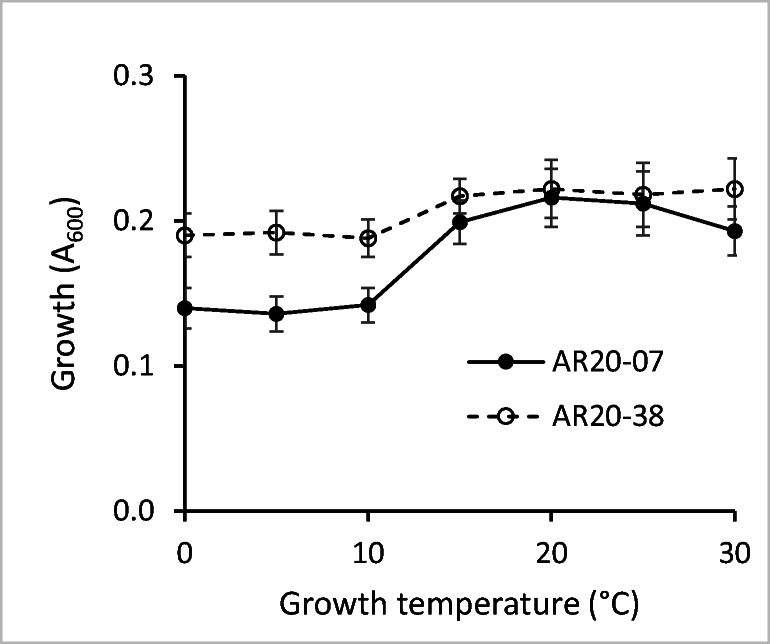
Effect of temperature on the utilization of LSS as sole carbon source by the two bacterial strains *Paraburkholderia aromaticivorans* AR20-38 and *Caballeronia udeis* AR20-07 (mean values ± SD of three replicates)

The LSS degradation potential of two strains, *Paraburkholderia aromaticivorans* AR20-38 (Poyntner et al. 2020) and *Caballeronia udeis* AR20-07 (both representatives of Betaproteobacteria, but belonging to different OTUs), was further tested in liquid culture. Both strains showed very similar growth patterns: good growth with LSS (2 g/L) was visible after 24 h at temperatures ranging from 5 to 30 °C; growth at 0 °C was 48 h delayed. The highest amount of biomass was produced at 15–30 °C; strain AR20-38 showed significantly better growth in the low temperature (0–10 °C) range than strain AR20-07 (Fig. 2).

Both strains utilized LSS amounts up to 15 g/L as sole carbon source. The higher the LSS concentration, the higher was the amount of biomass (i.e., biomass production was a linear function of LSS concentration); for example, both strains produced in the presence of 10 g/L LLS a ca. threefold higher biomass than in the presence of 2 g/L LLS. Independent of the LSS content, the stationary growth phase was reached after 48–72 h (data not shown).

### Utilization of organic pollutants as sole carbon source

The screening for utilization of one the organic pollutants n-hexadecane, diesel oil, phenol, and glyphosate as sole carbon sources on agar plates at 5 °C and 20 °C demonstrated that only a low fraction of the 68 tested strains was able to utilize one of the tested compounds at 5 °C (10 strains, 15%) or 20 °C (39 strains, 57%) (Table 2, Table S1).

Of the positive strains, substrate utilization was completely different at the two cultivation temperatures (Table 3). At 20 °C, the majority utilized diesel oil (44%) or glyphosate (36%) as the sole carbon source; at 5 °C, hexadecane (50%) or diesel oil (30%) was preferred. One strain (*Paraburkholderia aromaticivorans* AR20-38) utilized phenol very well for growth at 5 °C and 20 °C. Glyphosate was utilized by 21% at 20 °C, however, only by 1 strain (*Pedobacter cryoconitis* AR20-108) at 5 °C. C16 and diesel oil were preferentially utilized by members of Bacteroidetes and Betaproteobacteria at 20 °C, while glyphosate was mainly utilized by members of Bacteroidetes (Table S1).

None of the positive strains could utilize all four tested compounds at any of the test temperatures. The utilization of three out of the four compounds at 5 °C was noted by 1 strain (*Pedobacter cryoconitis* AR20-108), while 5 strains had this ability at 20 °C. The majority utilized one of the compounds at 5 °C (5 strains) and 20 °C (16 strains), respectively.

**Fig. 3:**
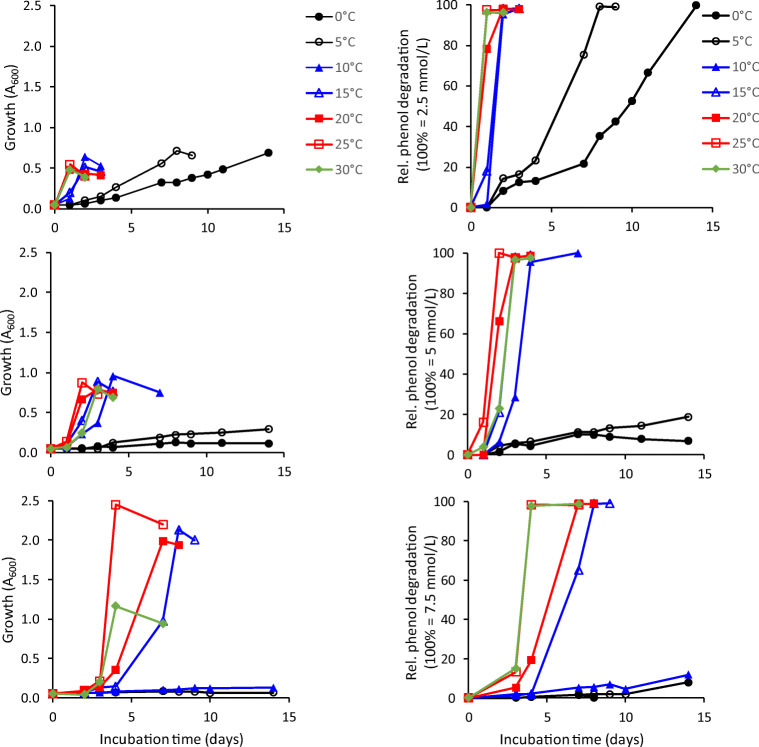
Effect of temperature and phenol concentration (top panels: 2.5 mmol/L; middle panels: 5 mmol/L; bottom panels: 7.5 mmol/L) on growth (left panels) and phenol degradation (right panels) by strain *Paraburkholderia aromaticivorans* AR20-38 (mean values of three replicates, SDs were ≤ 10%)

Strain AR20-38 (*Paraburkholderia aromaticivorans*; for characterization and identification of the strain see Poyntner et al. 2020) showed good growth both at 5 °C and 20 °C on agar plates containing 2.5 mmol/L phenol as the sole carbon source. Its phenol degradation potential was further tested in liquid culture. The evaluation of the effects of temperature (over the growth temperature range 0–30 °C) and phenol content demonstrated an increase in the lag phase with decreasing temperatures and increasing amounts of phenol (Fig. 3). An initial phenol content of 2.5 mmol/L resulted in complete phenol degradation after 1 day at 25–30 °C, after 2–3 days at 10–20 °C, and after 8 and 14 days at 5 °C and 0 °C, respectively. A phenol content of 5 mmol/L resulted in significantly reduced growth at 0–5 °C; complete phenol degradation occurred after 2 days at 25 °C, after 3 days at 15–20, and 30 °C and after 5 days at 10 °C. Biodegradation at lower temperatures was very low (19% at 5 °C, 7% at 0 °C). A similar pattern was observed at an initial phenol content of 7.5 mmol/L. The best growth was obtained at 25 °C, followed by 15–20 °C. Growth at 30 °C was already 50% lower. Full biodegradation occurred after 4 days at 25–30 °C and after 7–8 days at 15–20 °C. Growth and biodegradation were almost negligible at 0–5 °C (8-12%). 10 mmol/L phenol was not degraded at any of the temperatures.

## Discussion

The soil bacterial diversity at the Alpine coniferous forest site R has been reported (França et al. 2016). The majority of the 68 studied strains belonged to the phylum Proteobacteria, with a predominance of Beta- and Gammaproteobacteria, and a very low proportion of Alphaproteobacteria and Gram-positive representatives. The low presence of Gram-positive bacteria in Alpine soils has already been observed and can be attributed to their lower competitivity compared with Gram-negative bacteria, caused by, e.g., lower tolerance to freeze-thaw cycles and the promoting effect of plant roots towards Gammaproteobacteria (Margesin et al. 2009; França et al. 2016). However, a high abundance of Gram-positive representatives (mycobacteria) has been found in acidic boreal coniferous forest soils (Iivanainen et al. 1997).

In adaptation to the site-specific climate conditions, all strains, except one, were able to grow well over a broad temperature range from 5 to 25 °C. Representatives of the genera *Caballeronia* and *Collimonas* (Betaproteobacteria) were especially qualified for growth below 20 °C. These genera have already been described in cold soils (Männistö and Häggblom 2006; Zumsteg et al. 2013).

The majority of the organic matter in forest soils originates from plants. The screening for enzymes involved in the utilization of organic polymers present in litter demonstrated the widely distributed ability of the strains to degrade celluloses, xylan, and PGA both at low (0 °C) and moderate (20 °C) temperatures. Enzyme activities are required for the efficient litter decomposition and nutrient turnover in forest soils.

The main and most abundant cell wall polymer is cellulose and can be degraded by bacteria and fungi (Islam and Roy 2019). The cellulase complex consists of three major groups of cellulases (Yang et al. 2019). The high amount of strains in this study able to hydrolyze celluloses indicates that cellulases represent the main enzyme activities of the strains and demonstrates the bacterial role in litter decomposition. The presence of cellulose degraders at site R has been earlier observed (Margesin et al. 2016; Siles and Margesin 2017); however, in these studies, it was not possible to evaluate their response to temperature with regard to enzyme production. The almost comparable amount of (both Gram negative and Gram positive) CMC-hydrolyzing bacteria at low and moderate temperatures (5 °C and 20 °C) shown in this study indicates the wide distribution of this activity among soil bacteria, regardless of taxonomic affiliation or temperature. CMC is a water-soluble substrate that can be easily degraded by microorganisms. In contrast, the significantly higher proportion of strains able to hydrolyze the recalcitrant substrate micCell (avicelase) at 5 °C than at 20 °C points to the bacterial adaptation for the utilization of recalcitrant compounds at low temperatures, i.e., conditions prevailing at the site. As a consequence of the higher recalcitrance of coniferous litter, SOM at this site contains a high amount of aromatic compounds (Siles et al. 2017). Interestingly, strains tended to utilize either CMC or micCell, which points to the specialization of degraders for easily decomposable or recalcitrant compounds. Ladeira et al. (2015) hypothesized complex mechanisms of interaction within different types of cellulases. Generally, many cellulase producers at 5 °C belonged to Gammaproteobacteria. The production of polysaccharide-degrading enzymes by this class in cold environments has been reported (Jain and Krishnan 2017).

Xylan is a major polysaccharide in plant cell walls, and thus a quantitatively important carbon polymer in nature, found in hard wood, soft wood, and grasses. Xylan degradation is mainly conducted by microbial xylanases in nature; xylanases in cold Alpine soils have been described to be very diverse (Wang et al. 2010). Beside of cellulase, xylanase is the most important enzyme of primary litter degradation (Schinner et al. 1996). In our study, xylanase production was detected both at low and moderate temperature conditions, however, only by representatives of Bacteroidetes, and Beta- and Gammaproteobacteria. Xylanolytic representatives of these groups have also been described in Alpine tundra soil (Wang et al. 2010).

Pectic substances are ubiquitous in the plant kingdom. Microbial enzymes play a significant role for the decomposition and recycling of plant organic matter. The depolymerizing enzymes include polygalacturonidases and lyases, which cleave the glycosidic bonds by β-elimination (Margesin et al. 2005; Atanasova et al. 2018). The fraction of bacterial strains able to degrade PGA was lower than the fraction that utilized celluloses or xylan. This can be explained by the fact that pectic substances are not dominant in coniferous litter. The growth-inhibiting effects of xylan and PGA observed on agar plates in our study have already been reported and explained with the inhibitory or toxic effect of compounds released from these substrates during biodegradation (Rosenblatt et al. 2017; Brandon et al. 2018).

Another important biopolymer of plant cell walls is lignin. Lignin is the second most abundant organic substance in the world (Ganewatta et al. 2019). Due to its complex structure, comprising a matrix of phenolic and aliphatic substances, lignin is one of the most recalcitrant biopolymers (Bugg et al. 2011; Ganewatta et al. 2019). After plant death, lignin undergoes natural biodegradation by soil microorganisms, which results in the formation of SOM. In our study, a significantly higher number of strains utilized LSS as sole carbon source compared with lignin alkali. This can be explained by the water solubility and thus “easy” biodegradability of LSS (Silva et al. 2010), whereas lignin alkali is water insoluble and thus has a lower bioavailability (Chandra et al. 2008). Interestingly, all strains able to utilize lignin alkali belonged to the genus *Pseudomonas* (Gammaproteobacteria), while Burkholderiales dominated among LSS utilizers. Both groups have been described as efficient ligninolytic soil bacteria (Liu et al. 2019). There are a number of studies of bacteria able to degrade lignin, although earlier studies have focused on lignin breakdown by fungi (Bugg et al. 2011; Ganewatta et al. 2019).

Since lignin contains a high amount of aromatic and aliphatic compounds and a possible link between the degradation of aromatic compounds and lignin by soil bacteria has been observed (Bugg et al. 2011), we tested the ability of the bacterial strains to utilize organic pollutants as sole carbon source, to explore their potential for low temperature bioremediation. C16 and diesel oil were utilized especially by Bacteroidetes and Betaproteobacteria, both at low and moderate temperatures. Bacteroidetes were also mainly involved in the utilization of the pesticide glyphosate. Due to the ubiquity of hydrocarbons, bacteria capable of their utilization are present in every soil type, not only in contaminated ones (Welander 2005; Czarny et al. 2017). For example, bacteria capable of degrading a number of polyaromatic hydrocarbons were detected in non-contaminated mountain forest soil (Song et al. 2016). However, a higher amount of genes associated with biodegradation processes was found in contaminated soils (Czarny et al. 2017).

Phenolic compounds are highly toxic to microorganisms and can often cause the breakdown of wastewater treatment plants by inhibition of microbial growth even at low concentrations (< 2 mmol/L). Strain *Paraburkholderia aromaticivorans* AR20-38 utilized phenol over its whole growth temperature range (0–30 °C) and up to an amount of 7.5 mmol/L; its draft genome sequence has been described recently (Poyntner et al. 2020). The strain used the ortho type (C1,2D) of ring cleavage both at 5 °C and 20 °C. Ortho ring cleavage of catechol has also been observed with a representative of *Paraburkholderia phytofirmans* (Donos et al. 2017). The biodegradation potential of members of the genus *Paraburkholderia* for aromatic compounds has been reported (Li et al. 2017; Lee and Jeon 2018; Yuan et al. 2018). Community analyses showed that *Paraburkholderia* species degrade rarely n-hexadecane (Lee et al. 2019). The versatile metabolic capabilities of a representative of *Paraburkholderia aromaticivorans* (Lee et al. 2019) can also be assigned to strain AR20-38, since the strain utilizes lignin (this study) and a number of aromatic and polyaromatic hydrocarbons (catechol, phenol, naphthalene, phenanthrene) as sole carbon source (data not shown).

Catechol dioxygenase activities catalyze the ring cleavage of catechol. In our study, constitutively expressed catechol dioxygenase production was detected at low and moderate temperatures among a considerable part of the studied strains. Our data also indicated the clear preference of the ortho type (C1,2D) of ring cleavage for the oxidation of catechol. The predominance of the ortho-pathway has already been noted (Margesin et al. 2003, 2013). The additional presence of the meta type (C2,3D) of ring cleavage in some strains was especially noted at 5 °C. The constitutive expression of enzymes involved in the degradation of aromatic compounds may present an advantage for the immediate microbial response to forest litter.

In conclusion, the results reported in this study clearly demonstrate the degradation potential of culturable soil bacteria from Alpine coniferous forest site for organic polymers and pollutants. Representatives of Bacteroidetes were especially versatile. Thus, we proved the activity of genes that were found with the functional, however DNA-based, characterization of the soil microbial community (Siles and Margesin 2017). These results are useful to better understand the effect of changing temperatures on soil microorganisms involved in litter degradation and nutrient turnover. With an increase of soil temperatures in Alpine regions, as predicted due to global climate change (Gobiet et al. 2014), soil bacteria at the studied Alpine coniferous forest site could contribute to increased rates of SOM and litter degradation, which in turn could contribute to a positive feedback in climate warming. In addition, the demonstrated low temperature degradation potential and production of a range of enzymes could be useful for biotechnological application (Collins and Margesin 2019).

## Electronic supplementary material

ESM 1 (PDF 274 kb)
