# Supplementary material for: Culturable bacteria from an Alpine coniferous forest site: biodegradation potential of organic polymers and pollutants
Source: Folia Microbiol (Praha). 2020 Sep 25;66(1):87–98. doi: 10.1007/s12223-020-00825-1 (PMC7854452; doi:10.1007/s12223-020-00825-1)
Supplement: Supplementary file 1 — (PDF 274 kb) [file 12223_2020_825_MOESM1_ESM.pdf]

SUPPLEMENTARY MATERIAL (Table S1, Fig. S1)

Folia Microbiologica

Culturable bacteria from an Alpine coniferous forest site: biodegradation potential of organic polymers and pollutants

Tanja Berger[1], Caroline Poyntner[1], Rosa Margesin [1*]

* Corresponding author. E-mail address: rosa.margesin@uibk.ac.at

Table S1 Identification, growth temperature range on R2A agar, and effect of temperature (5°C and 20°C) on the production of enzymes for the degradation of organic polymers and the utilization of lignins and organic pollutants as sole carbon source by the 68 strains investigated.

0, no activity; 1, activity; 2, high activity; CMC, carboxymethylcellulose; micCell, microgranular cellulose; PGA, polygalacturonic acid; C1,2D, catechol-1,2-dioxygenase; C2,3D, catechol-2,3-dioxygenase; LSS, lignosulfonic acid; L alk, lignin alkali; GP, glyphosate

Strain	OTU	Phylum	Class	Species	Accession number	Growth temp. range (°C)	CMC 5°C	CMC 20°C	micCell 5°C	micCell 20°C	Xylan 5°C	Xylan 20°C	PGA 5°C	PGA 20°C	C1,2D 5°C	C1,2D 20°C	C2,3D 5°C	C2,3D 20°C	LSS 5°C	LSS 20°C	L alk 5°C	L alk 20°C	C16 5°C	C16 20°C	Diesel 5°C	Diesel 20°C	Phenol 5°C	Phenol 20°C	GP 5°C	GP 20°C
AR20-07	OTU01	Proteobacteria	Betaprote	*Caballeronia udeis*	MT280603	0-30	0	0	1	0	0	0	0	0	1	1	0	0	1	2	0	0	1	0	0	1	0	0	0	1
AR20-12	OTU01	Proteobacteria	Betaprote	*Caballeronia udeis*	MT758344	0-30	0	0	0	0	0	0	0	0	0	1	0	0	1	1	0	0	0	0	1	1	0	0	0	0
AR20-24	OTU01	Proteobacteria	Betaprote	*Caballeronia udeis*	MT758373	0-30	0	0	0	0	0	0	0	0	1	1	0	0	0	1	0	0	0	0	0	0	0	0	0	0
AR20-39	OTU01	Proteobacteria	Betaprote	*Caballeronia udeis*	MT761693	0-30	0	0	1	0	2	1	0	0	1	1	0	0	1	0	0	0	0	0	0	0	0	0	0	0
AR20-52	OTU01	Proteobacteria	Betaprote	*Caballeronia udeis*	MT762136	5-30	0	0	0	2	0	2	0	0	1	1	0	0	1	1	0	0	0	0	0	0	0	0	0	1
AR20-63	OTU01	Proteobacteria	Betaprote	*Caballeronia udeis*	MT762168	0-25	0	2	0	0	0	0	0	0	1	0	0	0	0	1	0	0	0	0	0	1	0	0	0	0
AR20-68	OTU01	Proteobacteria	Betaprote	*Caballeronia udeis*	MT762176	0-30	0	0	0	0	0	0	0	0	1	0	0	0	1	1	0	0	1	0	0	0	0	0	0	0
AR20-72	OTU01	Proteobacteria	Betaprote	*Caballeronia udeis*	MT762276	5-25	0	0	0	0	0	0	0	0	1	1	0	0	1	1	0	0	0	0	0	0	0	0	0	0
AR20-93	OTU01	Proteobacteria	Betaprote	*Caballeronia udeis*	MT762350	5-30	0	0	0	0	0	0	0	0	0	1	0	0	0	1	0	0	0	0	0	1	0	0	0	0
AR20-35	OTU02	Proteobacteria	Betaprote	*Collimonas pratensis*	MT758437	0-30	0	0	0	0	0	0	0	0	1	1	0	0	0	0	0	0	0	0	0	0	0	0	0	0
AR20-40	OTU02	Proteobacteria	Betaprote	*Collimonas pratensis*	MT761692	0-30	2	2	0	0	0	0	0	0	1	1	0	1	0	0	0	0	0	0	0	0	0	0	0	0
AR20-49	OTU02	Proteobacteria	Betaprote	*Collimonas pratensis*	MT761809	0-25	0	0	1	0	0	0	0	0	1	1	0	1	1	0	0	0	0	0	0	0	0	0	0	0
AR20-62	OTU02	Proteobacteria	Betaprote	*Collimonas pratensis*	MT762150	0-30	0	0	0	0	0	0	0	0	1	2	0	1	0	0	0	0	0	0	0	0	0	0	0	0
AR20-100	OTU02	Proteobacteria	Betaprote	*Collimonas pratensis*	MT762357	0-25	2	0	2	0	0	0	0	0	1	2	0	1	0	1	0	0	0	0	0	0	0	0	0	0
AR20-111	OTU02	Proteobacteria	Betaprote	*Collimonas pratensis*	MT281459	0-30	2	0	0	1	0	1	0	0	1	1	0	1	0	0	0	0	0	0	0	0	0	0	0	0
AR20-09	OTU03	Proteobacteria	Gammapr	*Luteibacter rhizovicinus*	MT758345	0-30	0	2	0	0	2	0	1	2	1	1	0	0	0	0	0	0	0	0	0	0	0	0	0	0
AR20-15	OTU03	Proteobacteria	Gammapr	*Luteibacter rhizovicinus*	MT758348	0-30	2	2	0	0	0	0	2	2	1	1	0	0	0	0	0	0	0	0	0	0	0	0	0	0
AR20-28	OTU03	Proteobacteria	Gammapr	*Luteibacter rhizovicinus*	MT758404	0-30	2	0	0	0	0	0	0	2	2	1	0	0	0	0	0	0	0	0	0	0	0	0	0	0
AR20-65	OTU03	Proteobacteria	Gammapr	*Luteibacter rhizovicinus*	MT281377	0-30	2	0	1	0	1	0	1	2	2	1	0	0	0	0	0	0	0	0	0	0	0	0	0	0
AR20-85	OTU03	Proteobacteria	Gammapr	*Luteibacter rhizovicinus*	MT762339	0-30	0	2	0	0	0	0	1	2	2	1	1	0	0	0	0	0	0	0	0	0	0	0	0	0
AR20-17	OTU04	Proteobacteria	Betaprote	*Paraburkholderia sediminicola*	MT280703	5-30	0	0	0	0	0	0	0	0	1	1	0	0	1	1	0	0	0	0	0	0	0	0	0	0
AR20-22	OTU04	Proteobacteria	Betaprote	*Paraburkholderia sediminicola*	MT762365	5-30	0	0	0	0	0	0	0	0	1	1	0	1	0	1	0	0	0	0	0	1	0	0	0	0
AR20-33	OTU04	Proteobacteria	Betaprote	*Paraburkholderia sediminicola*	MT758422	5-30	0	0	0	0	0	0	0	0	1	1	0	0	0	0	0	0	0	0	0	1	0	0	0	0
AR20-46	OTU05	Proteobacteria	Gammapr	*Dyella tabacisoli*	MT761699	5-30	0	0	0	0	0	0	0	0	1	1	0	0	0	0	0	0	0	0	0	0	0	0	0	0
AR20-53	OTU05	Proteobacteria	Gammapr	*Dyella tabacisoli*	MT281310	0-30	0	0	1	0	0	0	0	0	1	1	0	0	0	0	0	0	0	0	0	0	0	0	0	0
AR20-109	OTU05	Proteobacteria	Gammapr	*Dyella tabacisoli*	MT762365	5-30	0	0	0	0	0	0	0	0	1	1	0	0	0	0	0	0	0	0	0	0	0	0	0	0
AR20-32	OTU06	Proteobacteria	Betaprote	*Collimonas arenae*	MT758423	0-30	0	0	0	0	0	0	0	0	1	1	0	0	0	0	0	0	0	0	0	0	0	0	0	0
AR20-48	OTU06	Proteobacteria	Betaprote	*Collimonas arenae*	MT761800	0-30	0	0	2	0	0	0	0	0	1	1	0	0	0	0	0	0	0	0	0	0	0	0	0	0
AR20-82	OTU06	Proteobacteria	Betaprote	*Collimonas arenae*	MT281456	0-30	0	0	1	1	1	1	0	0	1	1	0	0	1	1	0	0	0	0	0	0	0	0	0	0
AR20-51	OTU07	Proteobacteria	Gammapr	*Rhodanobacter glycinis*	MT762126	0-25	2	0	0	0	0	0	0	0	1	1	0	0	0	0	0	0	0	0	0	0	0	0	0	0
AR20-88	OTU07	Proteobacteria	Gammapr	*Rhodanobacter spathiphylli*	MT762344	0-25	2	0	0	0	0	0	0	0	1	1	0	0	0	0	0	0	0	0	0	0	0	0	0	0
AR20-92	OTU07	Proteobacteria	Gammapr	*Luteibacter rhizovicinus*	KP899192	0-25	2	0	0	0	0	0	0	0	1	1	0	0	0	0	0	0	0	0	0	0	0	0	0	0
AR20-04	OTU08	Proteobacteria	Gammapr	*Pseudomonas migulae*	MT758264	0-30	0	0	2	0	1	0	1	1	1	1	0	0	0	0	0	0	0	0	0	0	0	0	0	0
AR20-84	OTU08	Proteobacteria	Gammapr	*Pseudomonas yamanorum*	MT762336	0-30	0	0	0	0	0	0	0	2	1	1	0	0	0	1	0	0	0	0	0	0	0	0	0	1
AR20-19	OTU09	Bacteroidetes		*Chryseobacterium aahli*	MT280791	0-30	2	2	2	2	0	2	0	0	2	2	1	1	0	1	0	0	0	1	0	1	0	0	0	1
AR20-38	OTU10	Proteobacteria	Betaprote	*Paraburkholderia aromaticivorans*	MT281269	5-30	0	0	1	0	2	0	0	0	1	1	0	0	1	2	0	1	0	0	0	0	1	2	0	0
AR20-36	OTU11	Proteobacteria	Betaprote	*Paraburkholderia sediminicola*	MT758438	5-30	0	0	0	0	0	1	0	0	1	1	0	0	0	0	0	0	0	0	0	0	0	0	0	1
AR20-71	OTU12	Proteobacteria	Gammapr	*Luteibacter rhizovicinus*	MT762203	0-30	2	0	1	0	0	0	0	0	1	1	0	0	0	0	0	0	0	0	0	0	0	0	0	0
AR20-75	OTU13	Proteobacteria	Betaprote	*Paraburkholderia sediminicola*	MT762290	0-30	0	0	0	0	0	0	0	0	1	1	0	0	0	1	0	0	0	0	0	0	0	0	0	0
AR20-25	OTU14	Bacteroidetes		*Flavobacterium psychroterrae*	MT758376	0-30	0	0	0	0	0	0	0	0	1	2	1	1	1	0	0	2	1	0	1	0	0	0	0	0
AR20-66	OTU15	Proteobacteria	Betaprote	*Variovorax ginsengisoli*	MT762175	5-30	0	2	0	0	0	0	0	0	1	1	0	0	0	0	0	0	0	0	0	0	0	0	0	0
AR20-108	OTU16	Bacteroidetes		*Pedobacter cryoconitis*	MT281458	0-30	2	2	2	1	2	1	0	0	1	2	1	1	1	2	0	0	1	2	1	0	0	0	1	1
AR20-87	OTU17	Proteobacteria	Gammapr	*Dyella japonica*	MT762343	5-30	0	0	0	0	0	0	0	0	1	1	0	0	0	0	0	0	0	0	0	0	0	0	0	1
AR20-70	OTU18	Proteobacteria	Gammapr	*Pseudomonas asplenii*	MT281378	0-30	0	0	0	0	0	0	0	0	0	0	0	0	1	1	1	2	0	0	0	0	0	0	0	0
AR20-110	OTU19	Proteobacteria	Alphaprote	*Rhizobium rhizogenes*	MT762361	0-30	0	0	0	0	0	0	0	0	0	1	0	0	0	1	0	0	0	0	0	0	0	0	0	0
AR20-47	OTU20	Proteobacteria	Gammapr	*Pseudomonas frederiksbergensis*	MT761801	0-30	0	0	0	0	0	0	0	0	1	1	0	0	0	0	0	1	0	0	0	0	0	0	0	0
AR20-01	OTU21	Actinobacteria		*Cellulomonas rhizosphaerae*	MT280698	5-25	2	2	0	0	0	0	0	0	1	1	0	0	0	0	0	0	0	0	0	0	0	0	0	0
AR20-58	OTU22	Proteobacteria	Alphaprote	*Mesorhizobium shangrilense*	MT762146	5-30	0	0	0	0	0	0	0	0	1	1	0	0	0	1	0	0	0	0	0	0	0	0	0	0
AR20-59	OTU23	Proteobacteria	Betaprote	*Paraburkholderia sediminicola*	MT762158	5-30	0	1	0	0	0	0	0	0	1	0	0	0	0	1	0	0	0	0	0	0	0	0	0	0
AR20-61	OTU24	Firmicutes		*Bacillus mycoides*	KP8999171	10-35	0	2	0	1	0	0	0	0	0	1	0	0	0	2	0	0	0	1	0	2	0	0	0	2
AR20-99	OTU25	Proteobacteria	Betaprote	*Paraburkholderia megapolitana*	MT762360	5-30	0	0	0	0	0	0	0	0	1	1	0	0	0	1	0	0	0	0	0	1	0	0	0	0
AR20-69	OTU26	Proteobacteria	Gammapr	*Pseudomonas brenneri*	MT762205	0-30	0	0	0	0	0	0	0	0	1	1	0	0	0	1	1	0	0	0	0	0	0	0	0	0
AR20-86	OTU27	Bacteroidetes		*Mucilaginibacter rubeus*	MT762338	5-30	0	2	1	2	0	1	0	0	1	1	1	1	0	1	0	0	0	1	0	0	0	0	0	1
AR20-97	OTU28	Bacteroidetes		*Mucilaginibacter jinjuensis*	KP899174	0-30	0	0	0	0	0	0	0	0	1	2	1	1	0	1	0	0	0	0	0	2	0	0	0	1
AR20-74	OTU29	Firmicutes		*Paenibacillus sinopodophylli*	MT762275	5-25	2	2	1	0	0	0	0	0	2	2	1	1	0	0	0	0	0	0	0	0	0	0	0	0
AR20-96	OTU30	Bacteroidetes		*Mucilaginibacter paludis*	MT762355	0-30	0	0	0	0	0	0	0	0	1	2	1	1	0	1	0	0	0	1	0	1	0	0	0	1
AR20-90	OTU31	Proteobacteria	Betaprote	*Burkholderia alpina*	MT762348	0-25	0	0	0	0	0	0	0	0	0	1	0	0	0	1	0	0	0	0	0	0	0	0	0	0
AR20-79	OTU32	Proteobacteria	Betaprote	*Paraburkholderia metrosideri*	MT762291	5-30	0	0	0	0	0	1	0	0	1	1	0	0	0	1	0	0	0	0	0	2	0	0	0	0
AR20-80	OTU33	Bacteroidetes		*Mucilaginibacter jinjuensis*	MT762298	0-30	0	0	0	0	0	0	0	0	2	2	1	1	0	0	0	0	0	1	0	1	0	0	0	1
AR20-57	OTU34	Proteobacteria	Betaprote	*Paraburkholderia bryophila*	MT762142	5-30	0	0	0	0	0	1	0	0	1	1	0	0	0	1	0	0	0	0	0	2	0	0	0	0
AR20-89	OTU35	Actinobacteria		*Diaminobutyricibacter tongyonger*	MT762349	5-30	2	2	0	0	0	0	0	0	1	1	0	0	0	0	0	0	0	0	0	0	0	0	0	0
AR20-02	OTU36	Bacteroidetes		*Pedobacter ginsenosidimutans*	MT758239	0-30	0	0	0	0	0	0	0	0	2	2	1	1	0	1	0	0	0	0	0	1	0	0	0	0
AR20-23	OTU37	Proteobacteria	Gammapr	*Pseudomonas laurentiana*	KP899169	0-35	0	0	0	0	0	0	0	1	1	1	0	0	0	0	0	2	1	0	0	0	0	0	0	0
AR20-30	OTU38	Bacteroidetes		*Mucilaginibacter angelicae*	MT758403	0-30	0	2	2	0	0	0	0	0	2	1	1	1	0	2	0	0	0	2	0	1	0	0	0	1
AR20-34	OTU39	Bacteroidetes		*Pedobacter ginsengiterrae*	MT758435	0-30	0	0	0	0	0	0	0	0	1	1	0	0	0	0	0	0	0	0	0	0	0	0	0	0
AR20-13	OTU40	Proteobacteria	Betaprote	*Variovorax boronicumulans*	MT758347	0-30	2	2	1	0	0	0	0	0	1	1	0	1	1	1	0	0	0	0	0	1	0	0	0	0
AR20-08	OTU41	Bacteroidetes		*Chryseobacterium soli*	MT758342	0-30	0	0	0	0	0	0	0	0	1	1	0	1	0	1	0	0	0	0	0	0	0	0	0	1
AR20-03	OTU42	Bacteroidetes		*Pedobacter ginsengisoli*	MT758241	5-30	0	2	1	1	0	0	0	0	2	2	1	1	0	0	0	0	0	0	0	0	0	0	0	0

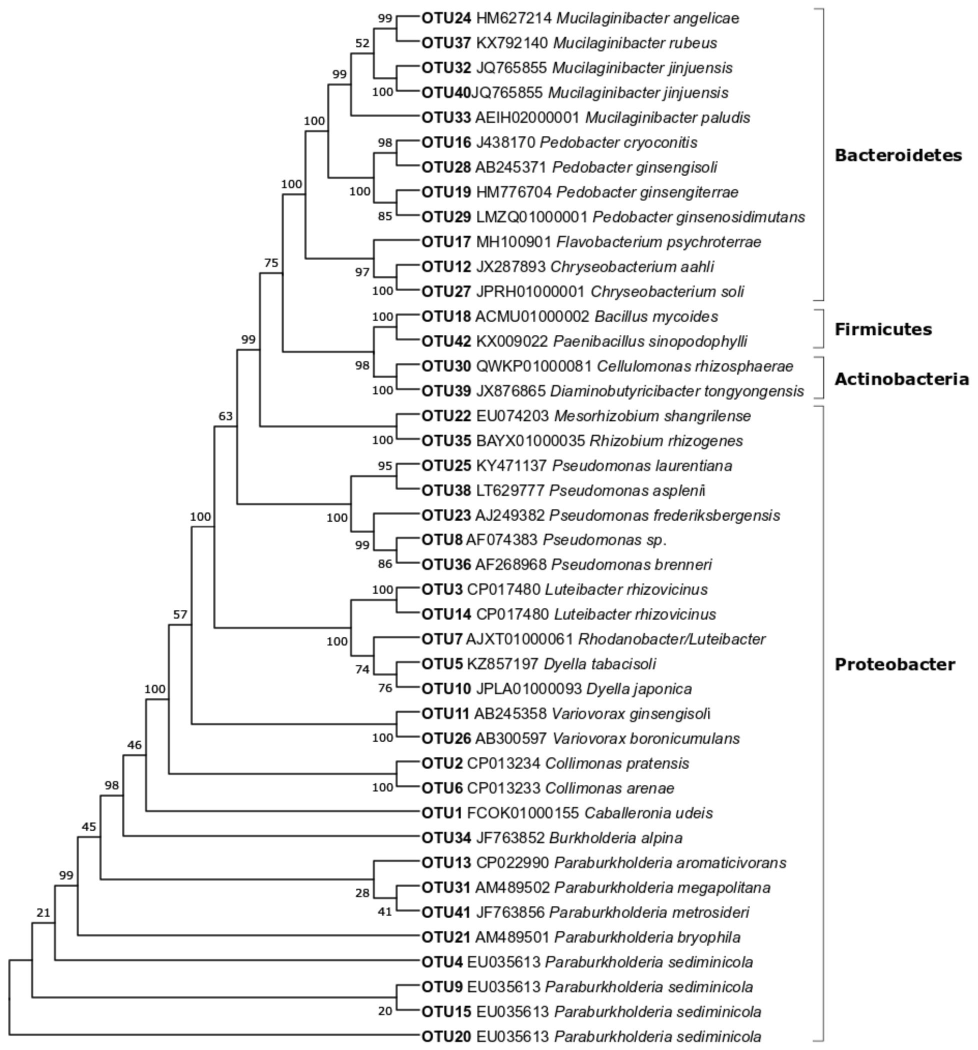

Fig. S1 Bootstrap consensus tree with the highest log likelihood (--11832.17) using Maximum Likelihood method and the Kimura 2-parameter model is shown. Numbers next to the branches show percentage of trees in which the associated taxa clustered and different phyla are marked on the right. OTUs, Accession numbers and reference species resulting from EzTazon-e Database are displayed. The tree is drawn to scale, with branch lengths measured in the number of substitutions per site
